# Immunogenic Evaluation of Ribosomal P-Protein Antigen P0, P1, and P2 and Pentameric Protein Complex P0-(P1-P2)_2_ of *Plasmodium falciparum* in a Mouse Model

**DOI:** 10.1155/2019/9264217

**Published:** 2019-09-12

**Authors:** Agnieszka Szuster-Ciesielska, Leszek Wawiórka, Dawid Krokowski, Nikodem Grankowski, Łukasz Jarosz, Urszula Lisiecka, Marek Tchórzewski

**Affiliations:** ^1^Department of Virology and Immunology, Maria Curie-Skłodowska University, Akademicka, 19 Lublin, Poland; ^2^Department of Molecular Biology, Maria Curie-Skłodowska University, Akademicka, 19 Lublin, Poland; ^3^Department of Epizootiology and Clinic of Infectious Diseases, University of Life Sciences, Głęboka, 30 Lublin, Poland

## Abstract

Malaria remains one the most infectious and destructive protozoan diseases worldwide. *Plasmodium falciparum*, a protozoan parasite with a complex life cycle and high genetic variability responsible for the difficulties in vaccine development, is implicated in most malaria-related deaths. In the course of study, we prepared a set of antigens based on P-proteins from *P. falciparum* and determined their immunogenicity in an *in vivo* assay on a mouse model. The pentameric complex P0-(P1-P2)_2_ was prepared along with individual P1, P2, and P0 antigens. We determined the level of cellular- and humoral-type immunological response followed by development of specific immunological memory. We have shown that the number of Tc cells increased significantly after the first immunization with P2 and after the second immunization with P1 and P0-(P1-P2)_2_, which highly correlated with the number of Th1 cells. P0 appeared as a poor inducer of cellular response. After the third boost with P1, P2, or P0-(P1-P2)_2_, the initially high cellular response dropped to the control level accompanied by elevation of the number of activated Treg cells and a high level of suppressive TGF-*β*. Subsequently, the humoral response against the examined antigens was activated. Although the titers of specific IgG were increasing during the course of immunization for all antigens used, P2 and P0-(P1-P2)_2_ were found to be significantly stronger than P1 and P0. A positive correlation between the Th2 cell abundance and the level of IL-10 was observed exclusively after immunization with P0-(P1-P2)_2_. An *in vitro* exposure of spleen lymphocytes from the immunized mice especially to the P1, P2, and P0-(P1-P2)_2_ protein caused 2-3-fold higher cell proliferation than that in the case of lymphocytes from the nonimmunized animals, suggesting development of immune memory. Our results demonstrate for the first time that the native-like P-protein pentameric complex represents much stronger immune potential than individual P-antigens.

## 1. Introduction

Despite numerous efforts, malaria is still one the most infectious and destructive protozoan diseases worldwide. According to the latest World Malaria Report (WHO, December 2017), in 2016, the estimated number of malaria cases reached 216 million with 5 million cases over the previous year, while the number of deaths was similar and reached approximately 445000. Among five protozoan species infecting humans, *Plasmodium falciparum* is responsible for most malaria-related deaths globally [1]. In the past decades, important progress in understanding the malaria life cycle and, especially, the availability of effective antimalarial drugs has been made; however, due to the development of drug-resistant *P. falciparum* strains, vaccination represents the most promising strategy for disease control. The main difficulties in vaccine development include the genetic variability of *P. falciparum*, which is a parasite survival strategy for immune system evasion, and its complex life cycle [2–4]. This is the reason why the dozens of *P. falciparum* antigens tested so far have turned out to be ineffective. One of the most extensively studied antigen was *P. falciparum* circumsporozoite protein 1 (CSP1), which became a promising vaccine component [5]. At present, the RTS,S/AS01 antigen, which targets the CSP1 of *P. falciparum* expressed at the preerythrocytic stage, was approved for use in Europe in 2015; however, the level of efficacy of RTS,S/AS01 is not sufficient to eliminate or eradicate malaria [5–8]. Besides improvement of the efficacy of the prototype vaccine, new approaches should also be developed. One of the promising antigens are *Plasmodium* sp. ribosomal P-proteins, which usually form an oligomeric complex with pentameric stoichiometry on the ribosome [9]. Immunological response against its scaffolding component, the P0 protein (uL10 according to the new nomenclature) [10], was defined as part of Naturally Acquired Immunity (NAI) against malaria [11]. The presence of specific anti-P0 antibodies in sera correlates with less severe clinical symptoms of malaria. Moreover, passive transfer of such antibodies or immunization with the P0 protein fragments conferred the malaria-specific protective properties in a murine model of the disease [12, 13]. In early trials, P0 was considered as a candidate for the vaccine development, but its immunogenicity turned out to be unsatisfactory. In general, the P0 protein belongs to a group of ribosomal proteins and is present within the cell as a component of the ribosomal particle, constituting a lateral ribosomal element, the so-called P-stalk structure [14]. In eukaryotes, the stalk has pentameric organization, P0-(P1-P2)_2_, with two P1-P2 dimers anchored to the bipartite alpha-helical region on the P0, which docks the whole P-complex to the ribosomal particle [15, 16]. It is generally accepted that the main function of this ribosomal structure is the activation of translational GTPases during each step of protein synthesis [17]. Multiplication of P-proteins, a phenomenon that is unique for ribosomal components, contributes to the fidelity of translation [18]. However, additional so-called extraribosomal function was ascribed for the P-protein, showing that this ribosomal proteins can be associated with numerous metabolic processes nonrelated to the ribosome activity, such as tumorigenesis [19, 20], apoptosis [21, 22], autophagy [23], and pathogenesis of autoimmunological diseases [24–26]. Interestingly, P-proteins were also associated with several protozoan infections including *Plasmodium* sp. The P0 protein was found on the *Plasmodium* sp. cell wall [27], whereas the P2 protein was localized on the surface of infected red blood cells at an early stage of the parasite development [28], underscoring the fact that not only P0 but also another component of the P-complex might have a prominent role in development of immunity against the malaria pathogen.

In this study, we aimed to analyze the immunogenicity of a new-generation antigen, based on ribosomal P-proteins from *Plasmodium falciparum*, namely, pentameric P0-(P1-P2)_2_, and compare it with individual P0, P1, and P2. Using a heterologous expression system in *E. coli*, several antigens were prepared, such as individual P1, P2, and P0 proteins. A P0-(P1-P2)_2_ pentameric complex was also produced, assembled in bacterial cells, and purified in a form reflecting the native features of the P-protein complex [29]. As a parasite-specific antigen, the Msp-1 protein was included as a reference. We evaluated the immune response against antigens *in vivo* with the mouse as an experimental model. Our results demonstrate for the first time that the native-like P-protein pentameric complex greatly enhances the immunogenic properties of the P-proteins and is critical for efficient development of cellular and humoral response as well as immunological memory.

## 2. Materials and Methods

### 2.1. Genetic Manipulations

DNA fragments carrying genes for the truncated form of P0 (amino acid residues 125-317) and the full length P1, P2, and Msp-1_19_ proteins were synthesized based on the sequences deposited in the *Plasmodium falciparum* 3D7 genome database (http://www.genedb.org/Homepage/Pfalciparum) with accession numbers PF11_0313, PF11_0043, PFC0400w, and PF3D7 0930300 for the P0, P1, P2, and Msp-1_19_ proteins, respectively. For all genetic manipulations, the DNA fragments containing the relevant genes were PCR amplified and introduced into a pT7-7 vector using specific restriction site EcoRI/BamHI. The DNA sequence encoding 6xHis-tag was introduced at the 5′-end of the genes, which resulted in fusion of the tag at the N-terminal part of the protein. All genetic constructs were verified by sequencing. In the case of the pentameric P0-(P1-P2)_2_ complex, a tricistronic expression cassette was used together with the pGEX4T-1 expression vector, as described previously [29].

### 2.2. Protein Expression and Purification

All recombinant proteins were expressed or coexpressed in *Escherichia coli* strain BL21(DE3) cells (Stratagene), as described previously [30]. In the case of the truncated form of P0_125-317_, further referred to as P0 or P1 recombinant proteins (which were present in cells as so-called inclusion bodies), the *E. coli* cells were disrupted by sonication, and the protein extract was solubilized in 8 M urea and purified by affinity chromatography on the Ni-NTA column (Sigma-Aldrich) in denaturing conditions, following the manufacturer's procedure. The obtained fractions of the P0 and P1 proteins eluted from the Ni-NTA resin were subsequently refolded by dialysis into buffer A (50 mM Tris-HCl pH 7.4, 100 mM NaCl, and 10 mM MgCl_2_). The recombinant P2 protein as well as Msp-1_19_ was purified by affinity chromatography on Ni-NTA resin (Sigma-Aldrich) in native conditions, according to the manufacturer's procedure, and dialyzed into buffer A. The P-proteins constituting the P0(P1-P2) complex were coexpressed in *E. coli* in a soluble form, with truncated P0 (fragments 197-317) fused to the GST protein. The complex was purified by affinity chromatography on the GST-trap column (Sigma-Aldrich), according to the manufacturer's instructions. The GST was removed by thrombin treatment, and the integrity of the complex was examined with nondenaturing mass spectrometry, as described previously [9]. The antigens were subjected to buffer exchange into buffer A immediately before formulation and mouse immunization.

### 2.3. Immunization of BALB/c Mice

Female 6-7 week-old BALB/c mice were purchased from Mossakowski Medical Research Centre, Polish Academy of Sciences (Warsaw, Poland), and housed at the Department of Animal Physiology (approved animal laboratory) under stable climatic and dietary conditions. All procedures involving the animals and handling thereof were conducted in accordance with the European Community Council Directive of 24 November 1986 (86/609/EEC) and Polish legislation acts concerning animal experimentation. The experimental protocols and procedures described in the study were approved by the Ethics Committee at the Medical University of Lublin. The mice were used in this study after 1 week of acclimatization.

Groups of 18 female BALB/c mice were immunized i.m. with 50 *μ*g of each protein antigen/mouse emulsified in complete Freund's adjuvant on day 1 and boosted on days 21 and 42 with the same amount of the antigen in incomplete Freund's adjuvant [31]. The control group was administered only with the adjuvants. Blood and sera were collected on days 14, 35, and 56 from six mice. For determination of cytokines (IL-2, IL-10, IFN-*γ*, and TGF-*β*) and specific antibodies, serum samples from each group were pooled. Biological material was also collected from untreated animals.

### 2.4. Cytotoxicity Assay

Spleen lymphocytes of untreated mice were incubated at 37°C for 24-48 h with different concentrations of the antigens (10-500/1000 *μ*g/ml). The toxicity of proteins was determined with the MTT assay according to the method described elsewhere [32]. The data from four independent experiments, each with eight separate lymphocyte suspensions, are presented as a percentage of control cell viability. Based on the cytotoxicity assay results, a nontoxic dose of the antigens was applied to *in vivo* studies (see [Supplementary-material supplementary-material-1] in the Supplementary Materials).

### 2.5. Proliferation Assay

Spleen lymphocytes were isolated two weeks after the last boost and incubated for 96 h with the protein that was used for immunization at 1 or 10 *μ*g/ml, based on the cytotoxicity assay results. PHA (5 *μ*g/ml) was used as a positive control. The toxicity of the proteins was determined with the BrdU (Roche Applied Science) proliferation assay. The stimulation index (defined as % viability of control cells lymphocytes of nonimmunized mice) of six individual lymphocyte suspensions performed in duplicate was calculated.

### 2.6. Flow Cytometry

Immunophenotypic analysis of peripheral blood cells was performed with an EPICS XL flow cytometer and the XL System II software (Beckman Coulter, USA). Double-color immunofluorescence assays were performed using a combination of PE- and FITC-conjugated rat anti-mouse monoclonal antibodies: IgG2a (MCA1212PE) and IgG2b (MCA1125PE) as a negative control, CD4FITC (MCA1107F), CD8RPE (MCS1260PE), CD30RPE (MCA1691PE), CD94RPE (MCS2288PE), CD25RPE (MCA1260PE) from AbD Serotec, and FoxP3PE (eBioscience).

### 2.7. Quantification of Anti-P0, Anti-P1, Anti-P2, Anti-P0-(P1-P2)_2_, and Anti-Msp-1_19_ Mouse Antibodies

MaxiSorp immunoplates (Nunc, Roskilde, Denmark) were coated with 100 *μ*l/well 1 *μ*g/ml of P0, P1, P2, P0-(P1-P2)_2_, or Msp-1_19_ proteins dissolved in 0.05 M carbonate-bicarbonate buffer (pH 9.6) (Sigma) overnight at 4°C. To titrate the sera, depending on the antibody class and subclass, 1 : 2, 1 : 3, or 1 : 4 serial dilutions in PBS-T were assayed (IgG and IgG1—1 : 64/1 : 104976; IgG2a—1 : 27/1 : 13122). Appropriate dilutions of secondary anti-mouse antibodies conjugated with alkaline phosphatase (100 *μ*l/well) were added: IgG (1 : 3000), IgM (Sigma), IgG1, and IgG2a (1 : 2000) (AbD Serotec). The serum dilution corresponding to a mean absorbance value of 0.1 was considered the end-point titer.

### 2.8. Cytokine Production by Lymphocytes

20 *μ*l of appropriate P-proteins was added (final protein concentration of 1 or 10 *μ*g/ml) to 3 × 10^6^ lymphocytes isolated from the untreated or immunized mouse spleen. The cells were incubated with the same protein that was used for immunization. Supernatants were collected after 24 h (TGF-*β*) or 72 h (IL-2, IL-10, and IFN-*γ*), and cytokines were assayed by ELISA with commercial kits (R&D Systems) and a microtiter plate reader (EMax, Molecular Devices Co.).

### 2.9. Statistical Analysis

The number of subjects included in this exploratory study was small. Significant findings, while useful for observation of trends in the data, are prone to error and must be confirmed in larger studies. Since the data were not normally distributed, nonparametric tests were used. For analysis of differences between two related samples, the Wilcoxon signed-rank test for significance was used. All study groups were compared with the Kruskal-Wallis test, followed by the Dunn multiple comparison test. Correlation analysis was performed using the Spearman rank correlation coefficient. A *P* value less than 0.05 was considered significant. Statistical analysis was carried out using Statistica software v12 (StatSoft Inc., Tulsa, OK, USA).

## 3. Results

### 3.1. Preparation of Antigens

**Figure 1:**
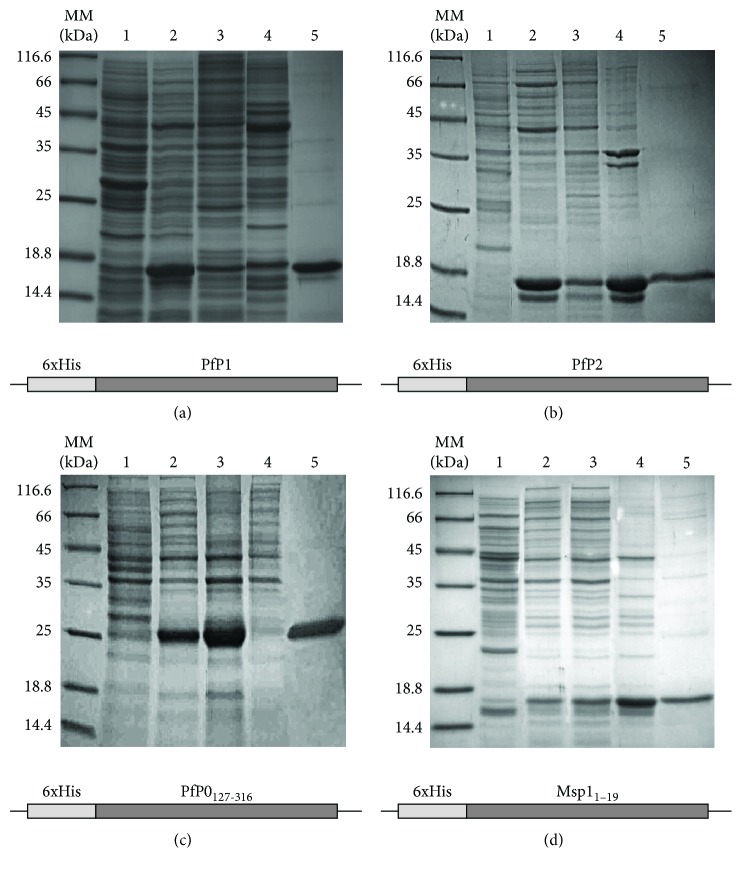
SDS-PAGE analysis of recombinant protein expression and purification. The individual P-proteins and the Msp-1_19_ control antigen were expressed in an *E. coli* BL21 (DE3) expression strain and purified using affinity chromatography on Ni-NTA resin. Lines: MM: molecular mass standards; lines 1 and 2: whole cell extract from *Escherichia coli* with an indicated expression vector without (-IPTG) and with (+IPTG) expression induction, respectively; lines 3 and 4: insoluble and soluble protein fractions, respectively; line 5: purified protein fraction eluted from the Ni column.

To compare the immunological response against various forms of *P. falciparum* P-antigens, a set of recombinant P-proteins, i.e., P1, P2, and P0, was prepared as 6xHis tagged forms, along with the Msp-1_19_ protein as a control. All proteins were efficiently expressed using an *E. coli* heterologous system. The recombinant P0 and P1 proteins were located in an insoluble protein fraction as inclusion bodies (Figures 1(a) and 1(c)). Both the P0 and P1 proteins were purified using nickel-affinity chromatography in denaturing conditions; subsequently, the proteins were refolded using the buffer exchange procedure into buffer deprived of a denaturing agent. The P2 and Msp1_19_ proteins were expressed in the soluble protein fraction (Figures 1(b) and 1(d)), and the purification was performed in native conditions. The pentameric complex of P-proteins, P0(P1-P2)_2_, was expressed in bacterial cells using a tricistronic expression cassette. All tested P1, P2, P0, and Msp1_19_ proteins displayed a purity level exceeding 95%, as quantified by SDS-PAGE (Figure 1). The *in vivo* assembled complex was purified by single-step affinity chromatography, in native conditions, using glutathione-affinity chromatography [29]. The complex was analyzed using the size-exclusion chromatography approach (SEC) and showed a single symmetrical peak indicating monodispersity of the sample; the SEC fractions used for subsequent immunization were analyzed with SDS-PAGE, and protein fraction displayed 95% purity ([Supplementary-material supplementary-material-1]).

### 3.2. T-cytotoxic Lymphocyte Response against Recombinant Ribosomal *P. falciparum* Antigens

**Figure 2:**
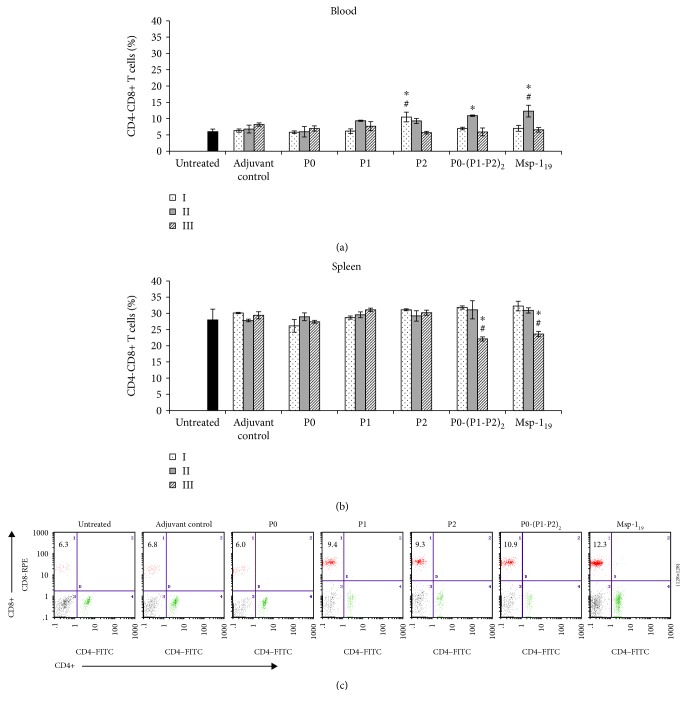
T-cytotoxic cell response after threefold immunization with the recombinant ribosomal *P. falciparum* proteins. Two weeks after each vaccination (denoted here as I, II, and III), blood (a) and spleen cells (b) were analyzed by flow cytometry for the number of CD4-CD8+ (Tc) lymphocytes. (c) Representative dot plots of CD4-CD8+ cells in peripheral blood of mice immunized twice with the P0, P1, P2, P0-(P1-P2)_2_, and Msp-1_19_ proteins, as well as in the adjuvant control and untreated animals. Numbers in the dot plot quadrants indicate the Tc cell percentage of total gated lymphocytes. Each data is presented as the mean ± S.E.M. of six mice. The differences were analyzed statistically with the Kruskal-Wallis test followed by the Dunn multiple comparison test. ^∗^Statistically significant in comparison to the corresponding boost in the adjuvant group, *P* ≤ 0.05. ^#^Differences are statistically significant between the indicated protein and the other proteins after the same boost number, *P* ≤ 0.05. We did not note significant differences between the untreated and adjuvant-treated animals.

The cellular response, which is critical in preerythrocytic stages of malaria infection and indispensable for elimination of intracellular liver-stage parasites, was quantified on the basis of changes in the number of T-cytotoxic effector cells (Tc, CD4-CD8+) in blood and spleen of immunized mice. Following the first immunization, only the P2 protein induced a significantly higher number (*P* ≤ 0.01) of blood Tc cells, which continuously decreased after the next immunizations (Figure 2(a)). Similar to the Msp-1_19_, also the P1 protein and P0-(P1-P2)_2_ complex enhanced Tc proliferation after the second immunization; however, the action of Msp-1_19_ was significantly (*P* ≤ 0.05) more effective (Figures 2(a) and 2(c)). The P0 and P1 antigens did not induce a significant increase in the Tc cell population. Irrespective of the proteins used, the blood CD8+ cell count decreased to a value comparable with the control after the last boosting. The number of splenic Tc cells in mice immunized with the P0, P1, and P2 proteins during threefold immunization remained unchanged and was comparable to that determined in adjuvant control animals. Two weeks after the last injection of P0-(P1-P2)_2_ and Msp-1_19_, the percentage of Tc cells significantly declined (*P* ≤ 0.05) (Figure 2(b)).

### 3.3. T-helper 1 Lymphocytes Enhance Cellular Response

**Figure 3:**
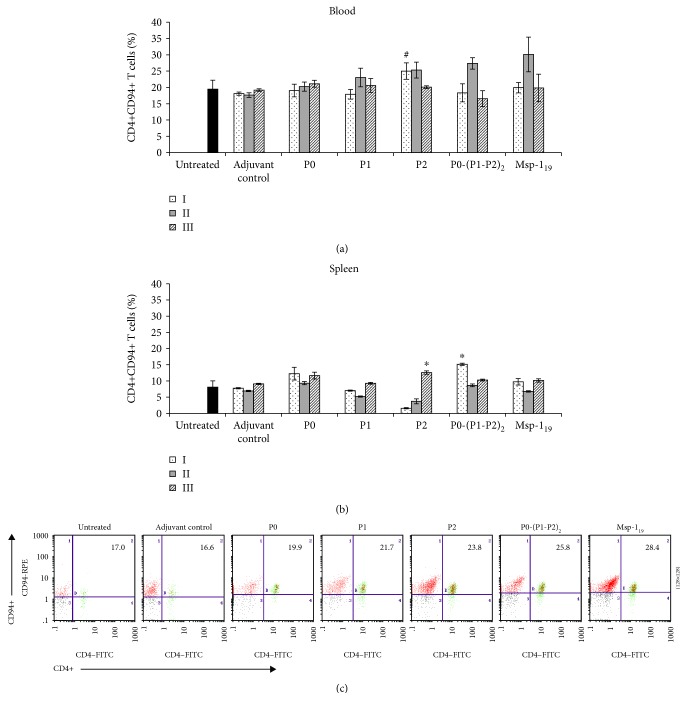
T-helper 1 cell response after threefold immunization with the recombinant ribosomal *P. falciparum* proteins. Two weeks after each vaccination (denoted here as I, II, and III), blood (a) and spleen cells (b) were analyzed by flow cytometry for the number of CD4+CD94+ (Th1) lymphocytes. (c) Representative dot plots of CD4+CD94+ cells in peripheral blood of mice immunized twice with the P0, P1, P2, P0-(P1-P2)_2_, and Msp-1_19_ proteins, as well as in the adjuvant control and untreated animals. Numbers in the dot plot quadrants indicate the Th1 cell percentage of total gated lymphocytes. Each data is presented as the mean ± S.E.M. of six mice. The differences were analyzed statistically with the Kruskal-Wallis test followed by the Dunn multiple comparison test. ^∗^Statistically significant in comparison to the corresponding boost in the adjuvant group, *P* ≤ 0.05. ^#^Differences are statistically significant between the indicated protein and the other proteins after the same boost number, *P* ≤ 0.05. We did not note significant differences between the untreated and adjuvant-treated animals.

To determine whether cellular response induced with recombinant proteins is supported by T-helper 1 (Th1, CD4+CD94+) lymphocytes, we analyzed their number in mouse blood and spleens. A significant increment (*P* ≤ 0.02) in the population of blood Th1 cells was noted after the first immunization with the P2 protein, which correlated (*r* = 0.69, *P* ≤ 0.0013) with the number of Tc cells and persisted after the second boost. Th1 response of similar intensity was achieved after the second immunization with P1 and P0-(P1-P2)_2_ and the reference antigen Msp-1_19_ (Figures 3(a) and 3(c)). Immunization with the P0 antigen had practically no impact on Th1 CD4+CD94+ abundance in any time during the course of the study. We noted a strong positive correlation between the number of Th1 and Tc lymphocytes in the peripheral blood of mice immunized with P1 (*r* = 0.62, *P* ≤ 0.005), P2 (*r* = 0.69, *P* ≤ 0.001), P0-(P1-P2)_2_ (*r* = 0.9, *P* ≤ 0.0003), and Msp-1_19_ (*r* = 0.91, *P* ≤ 0.0001) (see [Supplementary-material supplementary-material-1] in Supplementary Materials). In comparison with blood, the percentage of spleen Th1 cells was 2.5-fold lower in both untreated and adjuvant control mice. During immunization with the P2 and P0-(P1-P2)_2_ proteins, we noted the biggest boost-dependent alteration in the frequency of Th1 cells (Figure 3(b)). Two weeks after the first and the second immunizations with the P2 protein, we observed significant (*P* ≤ 0.002 compared with the adjuvant control) depletion in spleen Th1 cells, which statistically correlated with the increase in the number of blood lymphocytes (*r* = ‐0.91, *P* ≤ 0.001). Inversely, the first immunization with P0-(P1-P2)_2_ caused the highest frequency of Th1 cells among all proteins studied, which also negatively correlated with the numbers of blood Th1 (*r* = ‐0.82, *P* ≤ 0.01). Analysis of the frequencies of Th1 cells in peripheral blood and spleen revealed a significant negative correlation also in the case of P1 (*r* = ‐0.8, *P* ≤ 0.01) and Msp-1_19_ (*r* = ‐0.92, *P* ≤ 0.001).

### 3.4. T_reg_ Lymphocytes Regulate Cellular Response: Link with TGF-*β* and IL-10

**Figure 4:**
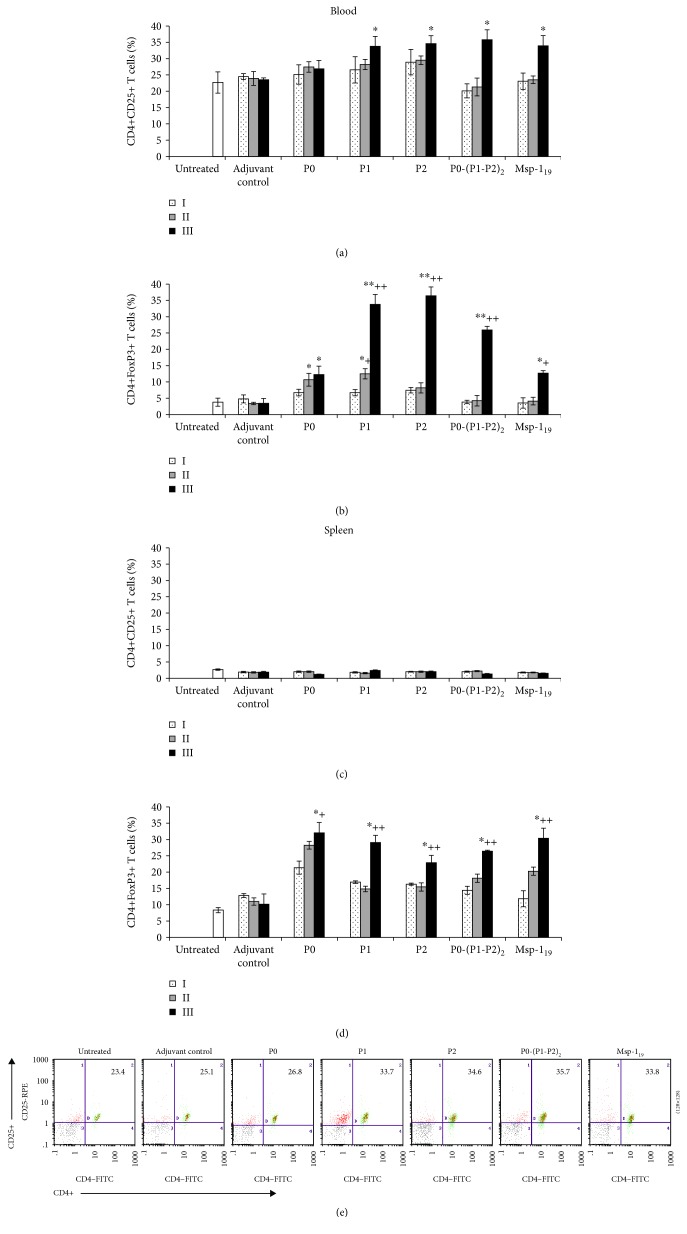
Regulatory immune response after threefold immunization with the recombinant ribosomal *P. falciparum* proteins. Two weeks after each vaccination (denoted here as I, II, and III), the number of blood (a, b) and spleen (c, d) CD4+CD25+ and CD4+FoxP3+ cells was determined by flow cytometry. (e) Representative dot plots of CD4+CD25+ cells in peripheral blood of mice immunized twice with the P0, P1, P2, P0-(P1-P2)_2_, and MSP-1_19_ proteins, as well as in the adjuvant control and untreated animals. Numbers in the dot plot quadrants indicate the CD4+CD25+ cell percentage of total gated lymphocytes. Each data is presented as the mean ± S.E.M. of six mice. The differences were analyzed statistically with the Kruskal-Wallis test followed by the Dunn multiple comparison test. ^∗^Statistically significant in comparison to the adjuvant group, ^∗^*P* ≤ 0.05, ^∗∗^*P* ≤ 0.005. ^+^Significantly different from the second immunization with the same protein, ^+^*P* ≤ 0.05, ^++^*P* ≤ 0.001.

The initially high cellular response against all antigens tested (except P0) dropping to the control level after the third boost raised a question about the development of active immunological suppression. Therefore, we evaluated the number of CD4+CD25+ and CD4+FoxP3+ cells in both blood and spleen of immunized mice. A significant (*P* ≤ 0.05) increase in blood CD4+CD25+ cells was observed after the last immunization with all antigen tested, except the P0 protein (Figures 4(a) and 4(e)). The differences between the antigens used became more pronounced when specific CD4+FoxP3+ T cells were evaluated. Such cells, regarded as activated regulatory T cells, were significantly more abundant (*P* ≤ 0.005) after the third immunization with P1, P2, or P0-(P1-P2)_2_ but not with the P0 or Msp1_19_ antigens (Figure 4(b)). While a negative correlation between CD4+CD25+ and Tc or Th1 cells was noted in almost all cases (see [Supplementary-material supplementary-material-1] in Supplementary Materials), statistically significant differences appeared during the comparison of the number of CD4+CD25+ and Th1 cells only after immunization with P2 (*r* = ‐0.82, *P* ≤ 0.00003), P0-(P1-P2)_2_ (*r* = ‐0.49, *P* ≤ 0.039), and Msp-1_19_ (*r* = ‐0.54, *P* ≤ 0.02). Similar correlations were observed between CD4+FoxP3+ and Tc or Th1 cells when P2 (*r* = ‐0.63, *P* ≤ 0.004 and *r* = ‐0.68, *P* ≤ 0.001, respectively), P0-(P1-P2)_2_ (*r* = ‐0.71, *P* ≤ 0.0008 and *r* = ‐0.65, *P* ≤ 0.003, respectively), or Msp-1_19_ (*r* = ‐0.56, *P* ≤ 0.01 and *r* = ‐0.48, *P* ≤ 0.04, respectively) were used (see [Supplementary-material supplementary-material-1] in Supplementary Materials). During the immunization, the number of spleen CD4+CD25+ remained at the same level; however, we noted progressive activation of T_reg_ that reached a maximum after the last boosting with all proteins (Figures 4(c) and 4(d)). Depending on the protein, these differences were statistically significant in the range of *P* ≤ 0.01 and *P* ≤ 0.05. Treatment with the adjuvant alone only resulted in a slight increase in CD4+FoxP3+ cells in the spleen of the control mice. To confirm the link between the activation of regulatory cells and the decrease in the cellular response, we performed Spearman analysis. It showed a strong negative correlation between spleen CD4+FoxP3+ and Tc cells when the following antigens were used: P2 (*r* = ‐0.72, *P* ≤ 0.027), P0-(P1-P2)_2_ (*r* = ‐0.81, *P* ≤ 0.007), and Msp-1_19_ (*r* = ‐0.76, *P* ≤ 0.017). In particular, a significant negative correlation between CD4+FoxP3+ and Th1 was noted only when P0-(P1-P2)_2_ was used (*r* = ‐0.85, *P* ≤ 0.003) (see [Supplementary-material supplementary-material-1] in Supplementary Materials).

**Figure 5:**
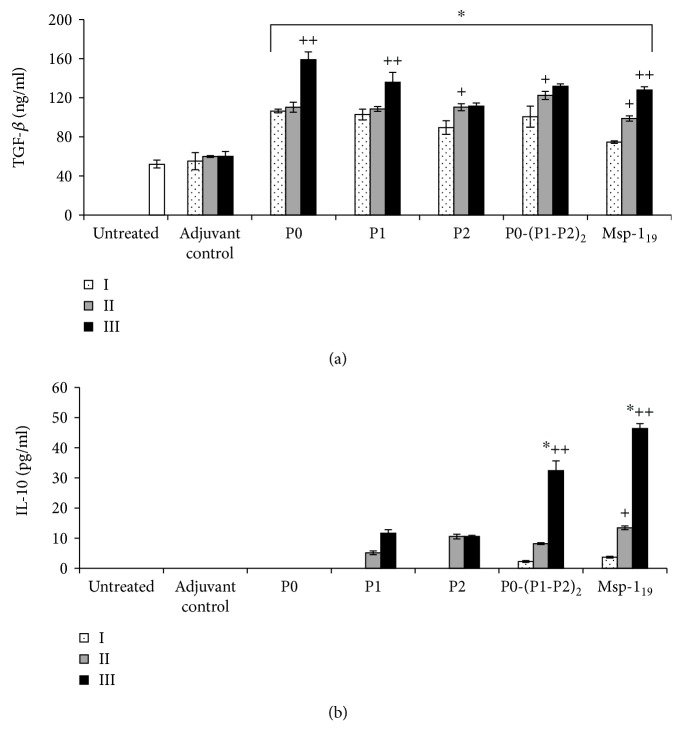
Increased production of TGF-*β* and IL-10 during immunization with the recombinant ribosomal *P. falciparum* proteins. Two weeks after each vaccination (denoted here as I, II, and III), TGF-*β* (a) and IL-10 (b) were measured by the ELISA method in sera of immunized mice as well as the adjuvant control and untreated animals. Results are the mean ± S.E.M. for three determinations of pooled sera from six mice in each group. The differences were analyzed statistically with the Kruskal-Wallis test followed by the Dunn multiple comparison test. ^∗^Statistically significant in comparison to the adjuvant group, ^∗^*P* ≤ 0.05. ^+^Significantly different from the second immunization with the same protein, ^+^*P* ≤ 0.05, ^++^*P* ≤ 0.001. (a) All the clamp-depicted bars have the same significance.

Previous studies indicated that FoxP3+ T_reg_ cells are derived from circulating CD4+CD25-FoxP3-T lymphocytes under stimulation with TGF-*β* and IL-10 [33]. To confirm this finding, we determined the level of cytokines in mouse sera two weeks after each immunization. As expected, all proteins induced significantly high production of TGF-*β* (Figure 5(a)), except for P0, which positively correlated with the number of blood-derived CD4+CD25+ and CD4+FoxP3+ cells (see [Supplementary-material supplementary-material-1] in Supplementary Materials). The highest serum IL-10 concentrations appeared only when P0-(P1-P2)_2_ or Msp-1_19_ were applied, reaching the maximum level after the last injection (Figure 5(b)), which was positively correlated with both the regulatory cell populations studied (see [Supplementary-material supplementary-material-1] in Supplementary Materials).

### 3.5. T-helper 2 Lymphocytes: Polarization of Immune Response

**Figure 6:**
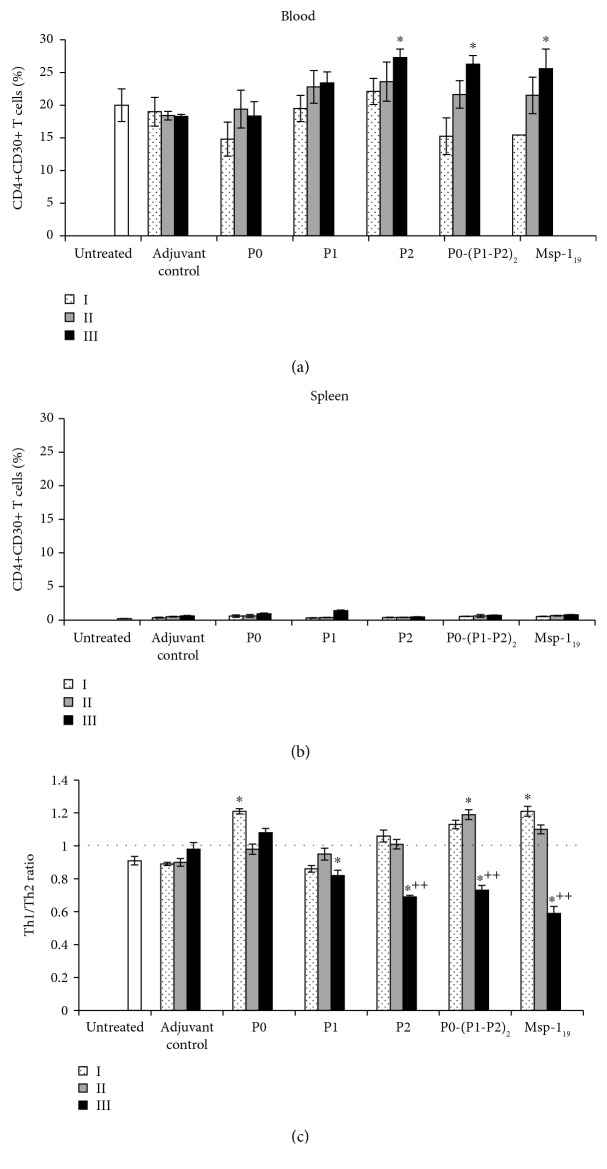
T-helper 2 cell response after threefold immunization with the recombinant ribosomal *P. falciparum* proteins. Two weeks after each vaccination (denoted here as I, II, and III), blood (a) and spleen cells (b) were analyzed by flow cytometry for the number of CD4+CD30+ (Th2) lymphocytes. The control groups consisted of adjuvant and untreated animals. Each data is presented as the mean ± S.E.M. of six mice. (c) Th1/Th2 ratio. The differences were analyzed statistically with the Kruskal-Wallis test followed by the Dunn multiple comparison test. ^∗^Statistically significant in comparison to the adjuvant group, ^∗^*P* ≤ 0.05. ^++^Significantly different from the second immunization with the same protein, *P* ≤ 0.001.

To assess whether our recombinant proteins are able to polarize immune response from the cellular to humoral direction, we determined the number of Th2 CD4+CD30+ cells in both blood and spleen of immunized mice (Figure 6). While the spleen Th2 population remained at the control level during all immunological studies, irrespective of the protein used (Figure 6(b)), we observed progressive expansion of blood Th2 cells. P2, P0-(P1-P2)_2_, and Msp-1_19_ were the most effective proteins and stimulated significant proliferation of Th2 cells after the third injection (*P* ≤ 0.01, in comparison with the first boost) (Figure 6(a)). Considering the Th1/Th2 ratio, we confirmed that initially P2, P0-(P1-P2)_2_, and Msp-1_19_ efficiently stimulated cellular response (ratio > 1), which was followed by enhancement of humoral response (ratio < 1) (Figure 6(c)). Th2 cells are the main source of IL-10, and, as expected, we noted a strong positive correlation between the number of Th2 blood lymphocytes and the serum IL-10 level when P2 (*r* = 0.65, *P* ≤ 0.05), P0-(P1-P2)_2_ (*r* = 0.82, *P* ≤ 0.006), or Msp-1_19_ (*r* = 0.81, *P* ≤ 0.007) were applied (see [Supplementary-material supplementary-material-1] in Supplementary Materials).

### 3.6. Production of Antibodies in response to Ribosomal P-protein Immunization

**Figure 7:**
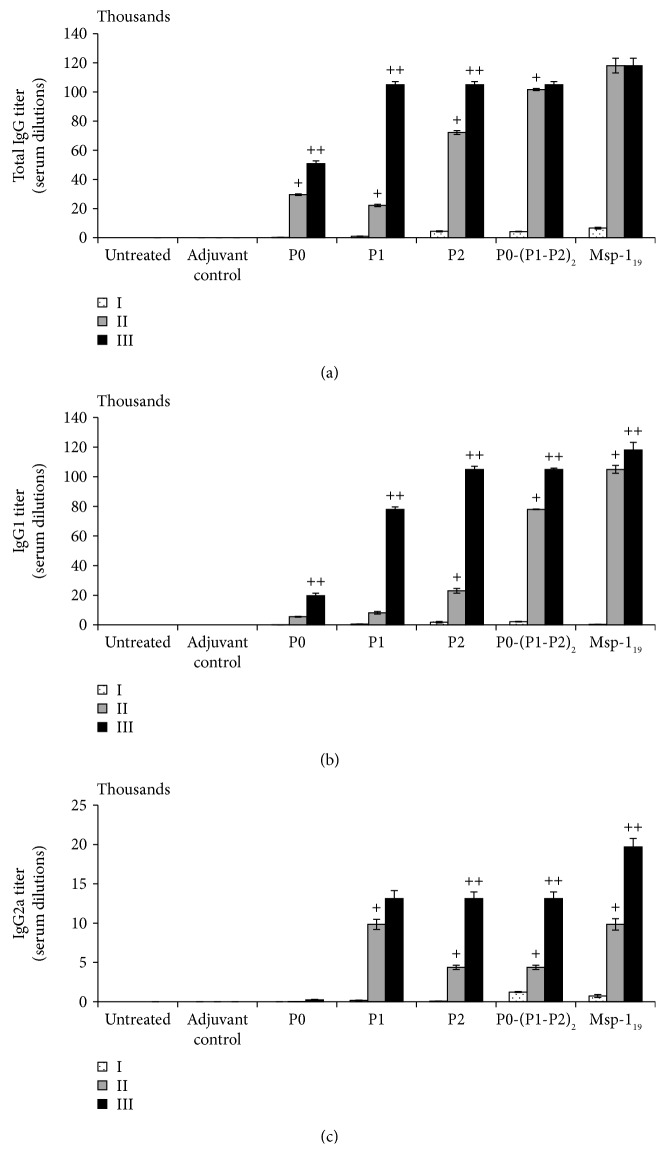
Humoral response after threefold immunization with the recombinant ribosomal *P. falciparum* proteins. Two weeks after each vaccination (denoted here as I, II, and III), the levels of antibodies were measured: total IgG (a), IgG1 (b), and IgG2a (c). The antibody titer was characterized as the reciprocal of the highest serum dilution factor corresponding to a mean absorbance value of 0.1. Each data is presented as the mean ± S.E.M. of six mice. The differences were analyzed statistically with the Kruskal-Wallis test followed by the Dunn multiple comparison test. Significantly different from (+) the first immunization or (++) the second immunization with the same protein, *P* ≤ 0.05.

**Figure 8:**
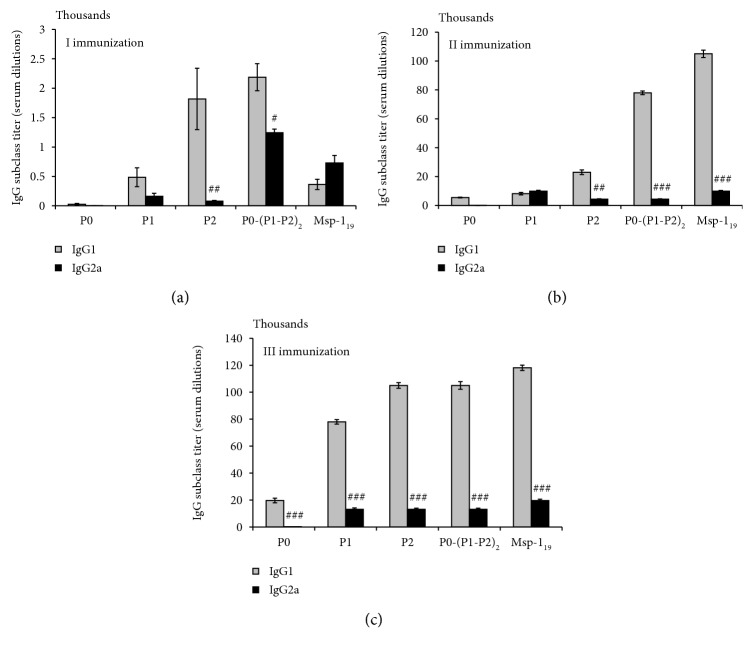
IgG1/IgG2a humoral response after threefold immunization with the recombinant ribosomal *P. falciparum* proteins. Predominance of Th1 response (IgG2a/IgG1 ratio > 1) was observed exclusively after the first Msp-1_19_ immunization (a), whereas all P-proteins gave a Th1 response (IgG2a/IgG1 ratio > 1) (a–c). The results are the mean ± S.E.M. for three determinations of pooled sera from six mice in each group. Statistical difference between the IgG1 and IgG2a levels in each study group was evaluated with the Wilcoxon signed-rank test; ^#^*P* ≤ 0.05, ^##^*P* ≤ 0.01.

Since the humoral immune response is known to be protective against *P. falciparum* infection and clinical malaria, we evaluated the ability of recombinant P-proteins to induce a set of immunoglobulins, total IgG, as well as its subpopulations IgG1 and IgG2a. The titers of specific IgG antibodies were increased after the immunizations with all the antigens tested, but the dynamics of production thereof varied. P2, P0-(P1-P2)_2_, and Msp-1_19_ induced high and statistically significant (*P* ≤ 0.01, in comparison with the first boost) IgG production already after the second immunization, while the P1 antigen caused IgG production comparable with that induced by the P2 and P-complex only after the last immunization (Figure 7(a)). Interestingly, the P0 antigen did not induce IgG production significantly, indicating its low immunogenicity. Additionally, following the Spearman analysis, we found significant positive correlations between the total IgG level and the blood Th2 numbers when the following proteins were used: P2 (*r* = 0.68, *P* ≤ 0.04), P0-(P1-P2)_2_ (*r* = 0.91, *P* ≤ 0.0006), and Msp-1_19_ (*r* = 0.88, *P* ≤ 0.001) (see [Supplementary-material supplementary-material-1] in Supplementary Materials). Apart from its regulatory role, IL-10 supports humoral response by inducing lymphocyte B proliferation and production of immunoglobulins. In our experiments, statistically significant correlations between the total IgG and IL-10 levels were found in the case of proteins P1 (*r* = 0.96, *P* ≤ 0.0004), P2 (*r* = 0.95, *P* ≤ 0.0006), P0-(P1-P2)_2_ (*r* = 0.69, *P* ≤ 0.037), and Msp-1_19_ (*r* = 0.66, *P* ≤ 0.049) (see [Supplementary-material supplementary-material-1] in Supplementary Materials). In particular, we noted a significant positive correlation between the IL-10 levels and the blood Th2 cell numbers after immunization of mice with P0-(P1-P2)_2_ (*r* = 0.82, *P* ≤ 0.006) and Msp-1_19_ (*r* = 0.82, *P* ≤ 0.007) (see [Supplementary-material supplementary-material-1] in Supplementary Materials). The immunoglobulin G1 (IgG1) isotype was predominant over IgG2a (with only one exception, as we added below) in a statistically significant manner, reflecting similar protein participation, as in the total IgG production (Figures 7(b) and 7(c)). Over the course of immunization, the level of IgG2a (an isotype promoted by Th1-like responses) increased more substantially than IgG1 (an isotype facilitated by Th2-like responses) in a nonsignificant manner only after the first immunization with the Msp-1_19_ protein (Figure 8(a)), whereas all P-proteins gave a Th1 response (IgG2a/IgG1 ratio > 1) (Figures 8(a)–8(c)).

### 3.7. Evidence of Cellular Memory Development

**Figure 9:**
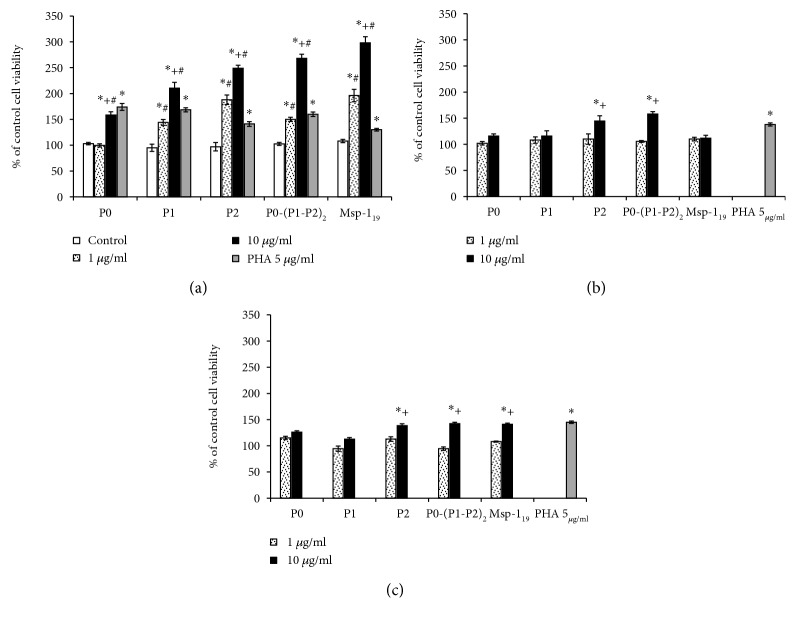
Analysis of cellular memory development. The analysis of proliferation of mouse spleen lymphocytes was performed in the presence of recombinant proteins (as described in Materials and Methods). (a) Spleen lymphocytes of threefold immunized mice incubated *in vitro* with the same protein that was used for vaccination. Spleen lymphocytes of the adjuvant control (b) and untreated (c) mice were incubated with recombinant proteins without earlier contact with them. In all groups, PHA (5 *μ*g/ml) was applied as a positive control. Each bar presents % of the viability of control cells (lymphocytes of nonimmunized mice) from six individual lymphocyte suspensions performed in duplicate. The results are expressed as the mean ± S.E.M. The differences were analyzed statistically with the Kruskal-Wallis test followed by the Dunn multiple comparison test. (a) ^∗^Statistically significant differences in comparison to the control cells (white bars) of immunized mice, ^∗^*P* ≤ 0.05. ^+^Significantly different in comparison to the lower protein concentration, *P* ≤ 0.05. ^#^Significantly different in comparison to the corresponding results obtained in the adjuvant control from (b), ^#^*P* ≤ 0.05. (b, c) ^∗^Differences are statistically significant in comparison to the protein-untreated cells of the control (untreated) animals. The viability of these cells was estimated at 100%. ^+^Significantly different in comparison to the lower protein concentration, ^∗^*P* ≤ 0.05.

To evaluate the immunological memory potential, we used *in vitro* exposure of spleen lymphocytes of immunized mice to recombinant proteins, measuring their ability to proliferate. We used a control mitogen, phytohemagglutinin (PHA), to check lymphocyte responsiveness. In comparison to the adjuvant-treated group, contact of lymphocytes isolated from threefold immunized animals with all recombinant proteins (except for P0) caused a two- to threefold (for P1, P2, P0-(P1-P2)_2_, and Msp-1_19_) increase in proliferation, which was dependent on the concentration of the proteins (Figure 9(a)). In this experiment, there were no significant differences between the untreated and adjuvant-injected animals (Figures 9(b) and 9(c)).

**Figure 10:**
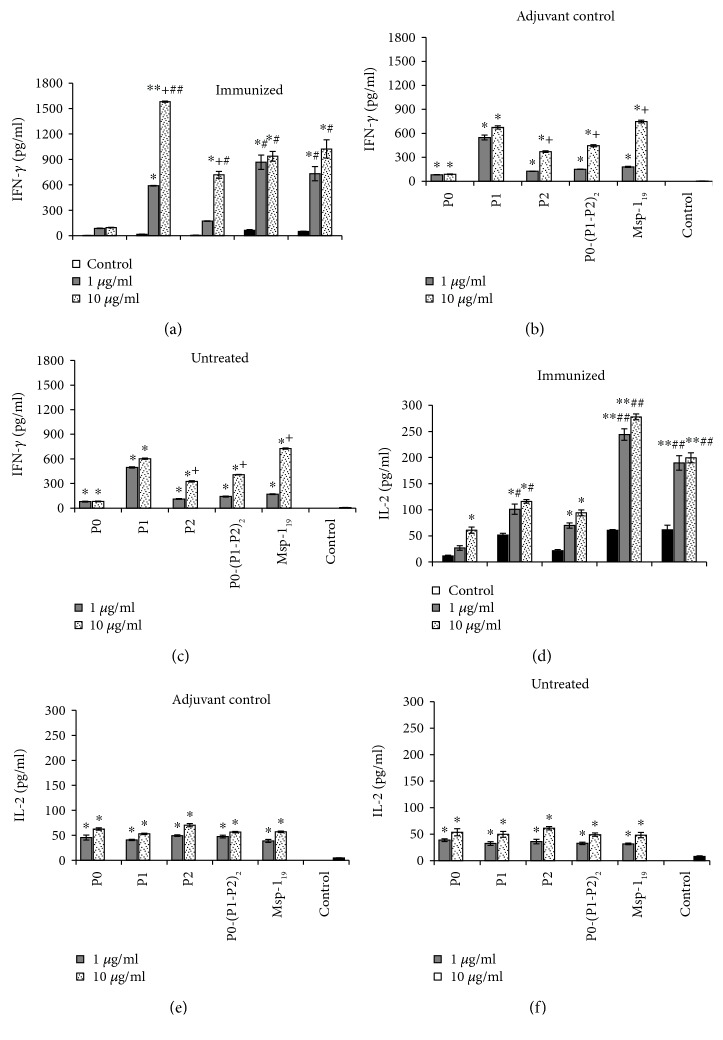
Analysis of cytokine production as cellular response following restimulation of spleen lymphocytes with recombinant proteins (*ex vivo* studies). IFN-*γ* (a–c) and IL-2 (d–f) production in spleen lymphocytes of immunized (a, d), control (adjuvant) mice (b, e), or control (untreated) mice (c, f). Following isolation, lymphocytes were incubated with 1 or 10 *μ*g/ml of proteins, and IFN-*γ* and IL-2 were measured in supernatants with the ELISA method after 72 h incubation. The results are the mean ± S.E.M. for three determinations of pooled sera from six mice in each group. Both IFN-*γ* and IL-2 concentrations in the sera of untreated animals were comparable to the adjuvant control mice (data not shown). The differences were analyzed statistically with the Kruskal-Wallis test followed by the Dunn multiple comparison test. ^∗^Differences are statistically significant in comparison to those shown in each control panel, ^∗^*P* ≤ 0.05, ^∗∗^*P* ≤ 0.005. ^+^Significantly different in comparison to the lower protein concentration, *P* ≤ 0.05. ^#^Significantly different in comparison to the corresponding results obtained in the adjuvant control, ^#^*P* ≤ 0.05, ^##^*P* ≤ 0.005.

Both Tc and Th1 cells are a source of IFN-*γ*, a cytokine with a critical role in immunity against *Plasmodium* sp. during liver-stage malaria. Although we did not detect IFN-*γ* in the sera of immunized mice, their spleen lymphocytes were able to produce significantly higher dose-dependent levels of this cytokine in response to the additional exposure to the antigen *in vitro* than the lymphocytes of the adjuvant and untreated animals (Figures 10(a)–10(c)). The data obtained in this experiment indicated that the P1 protein was the most effective, while P0-(P1-P2)_2_ and Msp-1_19_ acted similarly, inducing ca. 40% lower level of IFN-*γ* in comparison with P1. The P0 antigen did not induce significant production of IFN-*γ*. IL-2 was also found to be an important cytokine for preerythrocytic immunity, providing signals enhancing Tc proliferation, preventing their apoptosis, and inducing differentiation of a memory subpopulation. We did not detect IL-2 in the sera of immunized mice; however, their spleen lymphocytes produced over a fivefold higher level of IL-2 after the restimulation, especially with the P0-(P1-P2)_2_ antigen, than the spleen leukocytes of the control mice. The analogous response for individual P-proteins and Msp-1_19_ recall was less intensive (Figures 10(d)–10(f)), with the weakest IL-2 production after the P0 restimulation.

## 4. Discussion

The complex life cycle of *P. falciparum* and the variety of its mechanisms allowing evasion of the host immune response determine the unique requirements for malaria vaccine development. The current efforts are mainly focused on the specific antigens recognized as active elements of Naturally Acquired Immunity (NAI) against malaria, which could be potentially used for effective immunization. Here, we presented a new class of antigens based on a conserved *P. falciparum* ribosomal P-protein—a complex of P0, P1, and P2 proteins (P0-(P1-P2)_2_), which display high immunogenicity.

Previous observations have shown that people resistant to malaria symptoms have a high titer of specific antibodies recognizing the ribosomal P0 protein [11, 34, 35]. Moreover, the passive transfer of the anti-P0 antibodies or immunization of mice with the P0 polypeptide fragments conferred malaria-specific protection [13]. Additionally, the P2 protein, i.e., another component of the P-complex, was shown to be exported to the surface of infected red blood cells at an early parasite development stage [36, 37]. Specific anti-P2 IgG antibodies are able to cause parasite growth arrest at the onset of the nuclear division stage inside the erythrocyte, showing its unquestionable potential for vaccine development [37]. In the course of studies on the structure and function of ribosomal P-proteins, we have developed a new class of antigens based on the pentameric ribosomal P-protein complex, which was shown to have native-like properties of the P-complex in terms of biophysical and structural aspects [29]. Consequently, to acquire a complete view of the immunological properties of P-proteins, we have evaluated the immunogenic properties of individual components of the P-complex (namely, P0, P1, and P2 proteins) as well as a self-assembled P0(P1-P2)_2_ pentameric complex in a murine model. Additionally, we have used Msp1_1-19_, a well-characterized malaria antigen, as a reference to compare quantitatively the level of immunogenicity.

Effective protection against *P. falciparum* involves both cellular and humoral immunities, with antibody targeting free parasites at the blood stage and a cellular response important mainly in preerythrocytic stages [38]. The liver stage is the primary target for the vaccine-inducible T cell responses, because infected hepatocytes express a parasite's antigens that can be recognized and eliminated by CD8+ cells at an early stage of malaria development [39]. Interestingly, a significant increase in the population of Tc cells for P2, P0(P1-P2)_2_, and Msp1_1-19_ antigens was observed with the most rapid response induced by the P2 antigen (I immunization). The P0 and P1 proteins induced poor cellular-type immunological response, regardless of the number of boosting events. It is well documented that Th1 CD4+CD94+ cells support generation of liver stage-specific CD8+ T lymphocytes [40] mainly through secretion of cytokines: IL-2, a multifunctional cytokine, which can promote both effector T and B cell responses [41] and IFN-*γ* mediating the cytotoxic activity of CD8+ [5, 42, 43]. Interestingly, we observed dynamic changes in the number of spleen Th1 cells in response to P0-(P1-P2)_2_, P2, and Msp1_1-19_ but not to the P0 nor P1 proteins. However, the course of these changes differed: the P0-(P1-P2)_2_ protein complex and Msp1_1-19_ induced significant growth of the Th1 population after the second immunization, while the P2 protein had the same effect after the first immunization. Moreover, we noted a negative correlation between the spleen and blood Th1 cell content suggesting redistribution of active lymphocytes. These results are in line with the notion that T cells specific for the blood stage of the *Plasmodium* sp. life cycle are induced primarily in the spleen [44]. Importantly, the P0 antigen clearly represents the weakest cellular-type immunity inducing properties among the tested P antigens. At present, there is no information about the ability of infected hepatocytes to expose P-proteins of *P. falciparum*; hence, it is hard to estimate if the increased population of specific CD8+ cells that appeared in the mice after the immunization with our set of P-proteins could be efficient in eliminating infected hepatocytes.

The timing and intensity of different types of immune response development are crucial for the infection outcome. While an early proinflammatory response is required for reduction of parasite growth, anti-inflammatory mechanisms can prevent further organ damage [45]. We observed that the initially high cellular response against P0-(P1-P2)_2_, P2, and Msp1_1-19_ declined to the control level after the last immunization, which was negatively correlated with the increased number of T_reg_ (CD4+CD25+) cells and with the concentrations of both main suppressor cytokines TGF-*β* and IL-10. What is important, these cytokines are required for generation of activated regulatory cells (FoxP3+), which are key players in controlling excessive immune response [46]. In this context, both P0-(P1-P2)_2_ and Msp1_19_ proteins emerged to be the most efficient antigens. In contrast, we saw no significant induction of cellular-type response after the P0 stimulation, and there were no clear signs of active cellular-type response quenching. Since CD8+ cells with the support of Th1 cells have been shown to participate in the liver stage of malaria, it is clear that both Th2 and B cells are important components of the immune response against the blood stage of *Plasmodium* sp. infection [47]. Such sequentially biphasic CD4+ cell response appears during primary parasite infection [48]. In our studies, this immunological polarization was clearly seen as an increased number of blood Th2 CD4+CD30+ cells, in respect of the Th1 CD4+CD94+ population, developed after the third immunization with P0-(P1-P2)_2_, P2, and Msp-1_19_. It is tempting to speculate that the repeated immunizations with these antigens mimic immune response characteristic for the course of *Plasmodium sp*. infection. Supported by Th2 cells, generation of high antibody titers would prevent the invasion of erythrocytes by merozoites, enhance clearance of parasitized erythrocytes, or prevent their sequestration and thus the complications of malaria [49]. Considering the P-proteins, it has been shown that the anti-P0 antibodies can protect against malaria infection in the murine model [12, 13]. However, it should be noted that the protective effect developed after numerous repeated immunizations, and the application of the recombinant P0 protein induced a polyreactive low-titer humoral response, which was not able to inhibit parasite growth efficiently [50]. In line with those findings, we have shown that the P0 protein, in contrast to the other antigens tested, exhibits the weakest ability to induce antibody production. P2, P0-(P1-P2)_2_, and control Msp-1_19_ induced the highest IgG production already after the second immunization, while P1, after the last, third immunization. In a previous work, antibodies against the conserved C-terminal region common to P0/P1/P2 were preferentially produced by immunization with a human pentameric P0-(P1-P2)_2_ complex [51]. Moreover, monoclonal antibodies against C-terminal 16-mer peptide were the most effective inhibitor of *P. falciparum* invasion into erythrocytes [13]. The IgG1 and IgG2 isotypes in mice represent either Th2- or Th1-biased IgG isotypes, respectively, and the IgG1/IgG2 ratio is often used to evaluate the relative immune bias [52]. As in the case of Msp-1_19_, we detected that, after the second immunization, all P-antigens induced production of cytophilic IgG1 immunoglobulins, reflecting the Th2 response. Importantly, a strict positive correlation between the abundance of Th2 cells and the level of IL-10 (a cytokine supporting humoral-type response) was observed after the immunization with P0-(P1-P2)_2_ exclusively. This represents a very interesting finding, because cytophilic antiparasite immunoglobulins (those from the IgG1 and IgG3 subclasses) predominate in sera of infected people and often correlate with protection against malaria [53, 54]. We also obtained indirect evidence that immunization with the P-antigens elicited specific cellular-type immunological memory. An *in vitro* restimulation of spleen lymphocytes from the immunized mice with recombinant proteins caused their two- to threefold proliferation increase in the case of P1, P2, P0-(P1-P2)_2_, and Msp-1_19_. Again, P0-immunized spleen lymphocytes did not respond to the restimulation. Moreover, P1, P2, P0-(P1-P2)_2_, and Msp-1_19_, but not P0, induced production of high levels of IFN-*γ* (activating the cellular response indispensable for parasite elimination at the preerythrocytic stage), whereas only P1, P0-(P1-P2)_2_, and Msp-1_19_ induced IL-2 production (preventing Tc apoptosis, inducing the differentiation of memory subpopulation, and promoting effector T cell response) [41]. Consequently, the general picture emerging from our experiments shows that the pentameric form of the P-proteins, i.e., P0-(P1-P2)_2_, is a much stronger immunogen than the P0 protein alone, which has been frequently used as a primary antigen so far. In our experimental system, the P0 induced poor response at both the cellular and humoral levels. The possible explanation of such poor P0 immunogenicity observed in several reports could be related to the fact that the native molecular properties of the P0 protein were not taken into account. It is well documented that the P-proteins are present in eukaryotes as a component of the pentameric P0-(P1-P2)_2_ complex, the so-called ribosomal stalk complex on the 60S ribosomal subunit, conferring the functionality for the ribosomal particle [9, 15]. It has been shown that individual P-proteins strongly stimulate each other in acquisition of the native structure [55]; hence, their epitope presentation is critically dependent on the cooperative folding of all stalk components. Therefore, it seems rational that P0 alone does not acquire its native state, and immunization with a misfolded antigen can explain the low titer and the high level of polyreactivity of the specific anti-P0 antibodies observed previously [50]. Accordingly, in our studies, the immunization with P0 did not induce cellular-type immunity significantly and the humoral-type immunity was the weakest among the other P antigens tested. Additionally, contrary to P1, P2, P0-(P1-P2)_2_, and Msp-1_19_, the immunological response against P0 in the course of the immunizations did not follow the classical polarization from the cellular toward humoral type, and the P0 protein turned out to be a much weaker immunogen than P2, P0-(P1-P2)_2_, or even P1. Thus, the general immunogenic properties of P2 and especially the P0-(P1-P2)_2_ complex are comparable to the Msp-1_19_ level, which was used as a reference antigen in our experimental set-up. Further studies are required to check whether the specific anti-P0-(P1-P2)_2_ and anti-P2 immunological response is sufficient for inhibition of parasite growth and, consequently, prevents *Plasmodium* sp. infection.

## 5. Conclusion

Here, we presented a new class of antigens based on *P. falciparum* ribosomal P-proteins, P0, P1, P2, and their pentameric complex P0-(P1-P2)_2_. We found that these antigens induced efficient immune response in mice according to the following scenarios: (i) cellular response (which is critical in preerythrocytic stages of malaria and indispensable for elimination of intracellular liver-stage parasites), (ii) suppression of cellular response followed by polarization of immune response into the humoral type and production of specific antibodies (which play an important role in host defense and correlate with less severe clinical symptoms of malaria), and (iii) presence of immune memory. Our studies demonstrate for the first time that the recombinant *P. falciparum* ribosomal P-protein complex represents the most promising candidate for future development of a vaccine based on ribosomal P-proteins.

## Data Availability

The data underlying the findings of this study are included within the article and the supplementary information file.

## Supplementary Materials

Supplementary Materials Figure S1: cytotoxicity of recombinant ribosomal P-protein antigens. The viability of mouse spleen lymphocytes treated for 24-48 h with different concentrations of P0 (A), P1 (B), P2 (C), P0-(P1-P2)_2_ (D), and Msp-1_19_ (E) was determined with the MTT assay. The results are expressed as the mean ± S.E.M. of three independent experiments, each with eight separate cultures. ^∗^Significantly different from the 24 h incubation time, *P* ≤ 0.01 (Wilcoxon's signed-rank test). Figure S2: size-exclusion chromatography of the pentameric P-protein complex. Size-exclusion chromatography was performed using an analytical gel filtration column; the complex was eluted as a single symmetrical peak. Insert: SDS-PAGE analysis of protein fraction isolated from size-exclusion chromatography. Table S1: Th1 lymphocytes support cellular immune response. The Spearman correlation between the number of the Th1 and Tc lymphocytes in the peripheral blood of mice immunized with the P0, P1, P2, P0-(P1-P2)_2_, and MSP-1_19_ proteins and in the adjuvant control mice as well as the untreated control mice. Each group consisted of eighteen mice. Values of *P* ≤ 0.05 were considered significant. Table S2: regulation of cellular response (blood lymphocytes). The Spearman correlation between the numbers of CD4+CD25+ or CD4+FoxP3+ versus Tc and Th1 lymphocytes in the peripheral blood of mice immunized with the P0, P1, P2, P0-(P1-P2)_2_, and Msp-1_19_ proteins and in the adjuvant control mice as well as the untreated control mice. Correlations were calculated in each study group with eighteen mice. Values of *P* ≤ 0.05 were considered significant. Table S3: regulation of cellular response (spleen lymphocytes). The Spearman correlation between the numbers of CD4+FoxP3+ versus Tc and Th1 lymphocytes in cells isolated from the spleen of mice immunized with the P0, P1, P2, P0-(P1-P2)_2_, and Msp-1_19_ proteins and in the adjuvant control mice. Correlations were calculated in each study group with eighteen mice. Values of *P* ≤ 0.05 were considered significant. Table S4: interplay between regulatory blood lymphocytes and TGF-*β* or IL-10. The Spearman correlation between the numbers of serum TGF-*β* or IL-10 and CD4+CD25+ or CD4+FoxP3+ lymphocytes in the peripheral blood of mice immunized with the P0, P1, P2, P0-(P1-P2)_2_, and Msp-1_19_ proteins and in the adjuvant control mice. Correlations were calculated in each study group with eighteen mice. Values of *P* ≤ 0.05 were considered significant. The concentrations of circulating IL-10 levels in mice immunized with the P0 protein were below the detection threshold; therefore, calculation of Spearman correlations was not possible. Table S5: production of IL-10 is associated with increased Th2 lymphocyte numbers. The Spearman correlation between the numbers of serum IL-10 and Th2 (CD4+CD30+) lymphocytes in the peripheral blood of mice immunized with the P0, P1, P2, P0-(P1-P2)_2_, and Msp-1_19_ proteins and in the adjuvant as well as the untreated control mice. Correlations were calculated in each study group with eighteen mice. Values of *P* ≤ 0.05 were considered significant. The concentrations of circulating IL-10 levels in mice immunized with the P0 protein were below the detection threshold; therefore, calculation of Spearman correlations was not possible. Table S6: humoral immune response support. The Spearman correlation between the IgG levels and the abundance of Th2 lymphocytes in the peripheral blood of mice immunized with the P0, P1, P2, P0-(P1-P2)_2_, and Msp-1_19_ proteins. For IgG determination, sera in each mouse group were pooled (*n* = 9). Values of *P* ≤ 0.05 were considered significant. Table S7: IL-10 promotes humoral response. The Spearman correlation between IgG levels versus IL-10 serum concentrations and Th2 lymphocyte numbers in the peripheral blood of mice immunized with the P0, P1, P2, P0-(P1-P2)_2_, and MSP-1_19_ proteins and in the adjuvant control mice as well as the untreated control mice. For measurements of both IgG and IL-10, sera in each mice group were pooled (*n* = 9). P0 did not induce detectable production of serum IL-10. Values of *P* ≤ 0.05 were considered significant.
