# Supplementary material for: Immunogenic Evaluation of Ribosomal P-Protein Antigen P0, P1, and P2 and Pentameric Protein Complex P0-(P1-P2)2 of Plasmodium falciparum in a Mouse Model
Source: J Immunol Res. 2019 Sep 12;2019:9264217. doi: 10.1155/2019/9264217 (PMC6757288; doi:10.1155/2019/9264217)
Supplement: Supplementary Materials — Figure S1: cytotoxicity of recombinant ribosomal P-protein antigens. The viability of mouse spleen lymphocytes treated for 24-48 h with different concentrations of P0 (A), P1 (B), P2 (C), P0-(P1-P2)2 (D), and Msp-119 (E) was determined with the MTT assay. The results are expressed as the mean ± S.E.M. of three independent experiments, each with eight separate cultures. ∗Significantly different from the 24 h incubation time, P ≤ 0.01 (Wilcoxon's signed-rank test). Figure S2: size-exclusion chromatography of the pentameric P-protein complex. Size-exclusion chromatography was performed using an analytical gel filtration column; the complex was eluted as a single symmetrical peak. Insert: SDS-PAGE analysis of protein fraction isolated from size-exclusion chromatography. Table S1: Th1 lymphocytes support cellular immune response. The Spearman correlation between the number of the Th1 and Tc lymphocytes in the peripheral blood of mice immunized with the P0, P1, P2, P0-(P1-P2)2, and MSP-119 proteins and in the adjuvant control mice as well as the untreated control mice. Each group consisted of eighteen mice. Values of P ≤ 0.05 were considered significant. Table S2: regulation of cellular response (blood lymphocytes). The Spearman correlation between the numbers of CD4+CD25+ or CD4+FoxP3+ versus Tc and Th1 lymphocytes in the peripheral blood of mice immunized with the P0, P1, P2, P0-(P1-P2)2, and Msp-119 proteins and in the adjuvant control mice as well as the untreated control mice. Correlations were calculated in each study group with eighteen mice. Values of P ≤ 0.05 were considered significant. Table S3: regulation of cellular response (spleen lymphocytes). The Spearman correlation between the numbers of CD4+FoxP3+ versus Tc and Th1 lymphocytes in cells isolated from the spleen of mice immunized with the P0, P1, P2, P0-(P1-P2)2, and Msp-119 proteins and in the adjuvant control mice. Correlations were calculated in each study group with eighteen mice. Values of P ≤ 0.05 [file 9264217.f1.pdf]

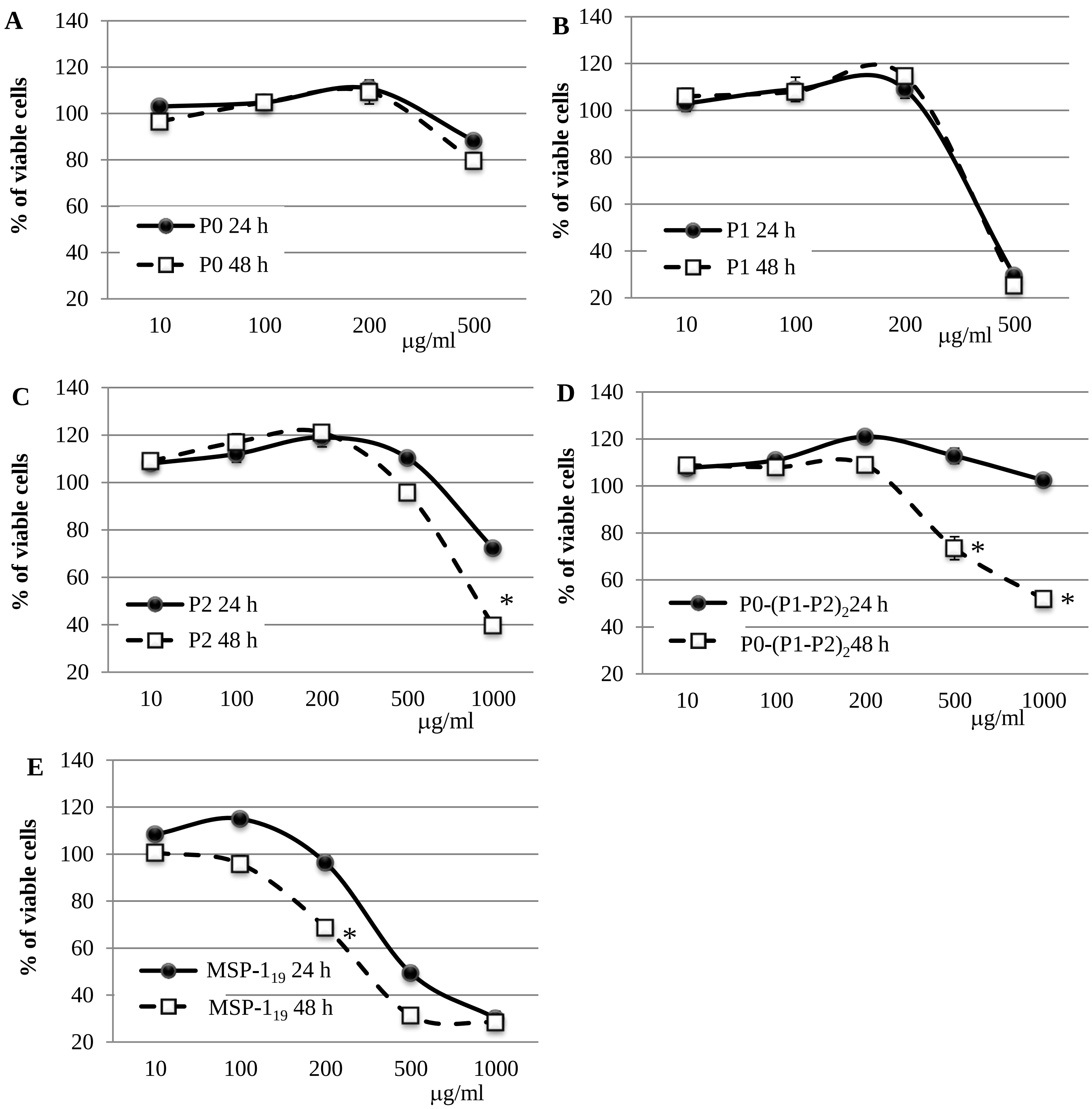

Figure S1.

Cytotoxicity of recombinant ribosomal P-protein antigens. The viability of mouse spleen lymphocytes treated for 24-48h with different concentrations of P0 (A), P1 (B), P2 (C), P0-(P1-P2)$_2$ (D), and Msp-1$_{19}$ (E) was determined with the MTT assay. The results are expressed as the mean ± S.E.M. of three independent experiments, each with eight separate cultures. *- significantly different from the 24h incubation time, $P \leq 0.01$ (Wilcoxon's signed rank test).

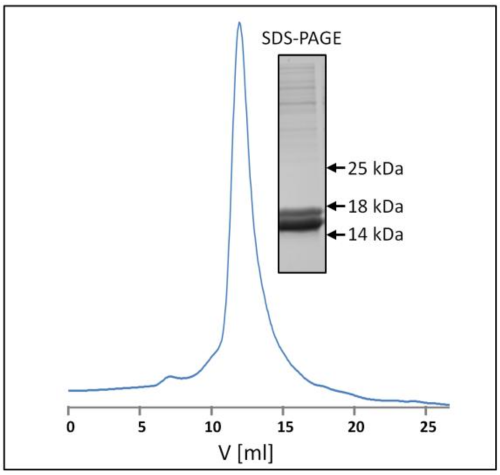

Figure S2.

Size-exclusion chromatography of the pentameric P-protein complex. Size-exclusion chromatography was performed using an analytical gel filtration column; the complex was eluted as a single symmetrical peak. Insert; SDS-PAGE analysis of protein fraction isolated from size-exclusion chromatography.

Mice treated with	Th1 versus Tc	
	r	*P*
P0	0.14	0.57
P1	0.62	0.005
P2	0.69	0.001
P0-(P1-P2)$_2$	0.92	0.0003
Msp-1$_{19}$	0.91	0.0001
adjuvant control mice	0.14	0.58
untreated control mice	0.22	0.41

Table S1.

Th1 lymphocytes support cellular immune response. The Spearman correlation between the number of the Th1 and Tc lymphocytes in the peripheral blood of mice immunized with the P0, P1, P2, P0-(P1-P2)$_2$, and MSP-1$_{19}$ proteins and in the adjuvant control mice as well as the untreated control mice. Each group consisted of eighteen mice. Values of $P \leq 0.05$ were considered significant.

.

Protein	CD4+CD25+ versus:				CD4+FoxP3+ versus:			
	Tc		Th1		Tc		Th1	
	r	*P*	r	*P*	r	*P*	r	*P*
P0	-0.046	0.85	0.13	0.59	0.17	0.49	0.2	0.42
P1	-0.15	0.55	-0.13	0.56	0.31	0.2	0.3	0.22
P2	-0.38	0.11	-0.82	0.00003	-0.63	0.004	-0.68	0.001
P0-(P1-P2)$_2$	-0.46	0.053	-0.49	0.039	-0.71	0.0008	-0.65	0.003
Msp1$_{19}$	-0.43	0.07	-.054	0.02	-0.56	0.01	-0.48	0.04
adjuvant control mice	0.28	0.24	-0.079	0.75	0.12	0.37	-0.1	0.62
untreated control mice	0.3	0.11	0.1	0.66	0.26	0.21	0.15	0.54

Table S2.

Regulation of cellular response (blood lymphocytes). The Spearman correlation between the numbers of CD4+CD25+ or CD4+FoxP3+ versus Tc and Th1 lymphocytes in the peripheral blood of mice immunized with the P0, P1, P2, P0-(P1-P2)$_2$, and Msp-1$_{19}$ proteins and in the adjuvant control mice as well as the untreated control mice. Correlations were calculated in each study group with eighteen mice. Values of $P \leq 0.05$ were considered significant.

Protein	CD4+FoxP3+ versus:			
	Tc		Th1	
	r	*P*	r	*P*
P0	0.45	0.22	0.31	0.4
P1	-0.016	0.96	-0.26	0.48
P2	-0.72	0.02	0.38	0.3
P0-(P1-P2)$_2$	-0.81	0.007	-0.85	0.003
Msp-1$_{19}$	-0.76	0.017	-0.4	0.28
adjuvant control mice	0.02	0.94	0.36	0.32
untreated control mice	0.12	0.84	0.22	0.61

Table S3.

Regulation of cellular response (spleen lymphocytes). The Spearman correlation between the numbers of CD4+FoxP3+ versus Tc and Th1 lymphocytes in cells isolated from the spleen of mice immunized with the P0, P1, P2, P0-(P1-P2)$_2$, and Msp-1$_{19}$ proteins and in the adjuvant control mice. Correlations were calculated in each study group with eighteen mice. Values of $P \leq 0.05$ were considered significant.

Mice treated with	TGF-β versus:				IL-10 versus:			
	CD4+CD25+		CD4+FoxP3+		CD4+CD25+		CD4+FoxP3+	
	r	*P*	r	*P*	r	*P*	r	*P*
P0	-0.2	0.59	0.5	0.07	-	-	-	-
P1	0.35	0.34	0.7	0.01	0.61	0.076	0.81	0.007
P2	0.7	0.03	0.8	0.001	0.44	0.23	0.72	0.025
P0-(P1-P2)$_2$	0.82	0.005	0.51	0.01	0.66	0.049	0.5	0.16
Msp-1$_{19}$	0.88	0.002	0.55	0.05	0.45	0.21	0.55	0.17
adjuvant control mice	-0.15	0.49	0.1	0.61	0.11	0.33	0.2	0.21
untreated control mice	-0.1	0.59	-0.19	0.69	0.17	0.84	0.11	0.66

Table S4.

Interplay between regulatory blood lymphocytes and TGF-β or IL-10. The Spearman correlation between the numbers of serum TGF-β or IL-10 and CD4+CD25+ or CD4+FoxP3+ lymphocytes in the peripheral blood of mice immunized with the P0, P1, P2, P0-(P1-P2)$_2$, and Msp-1$_{19}$ proteins and in the adjuvant control mice. Correlations were calculated in each study group with eighteen mice. Values of $P \leq 0.05$ were considered significant.

- the concentrations of circulating IL-10 levels in mice immunized with the P0 protein were below the detection threshold; therefore, calculation of Spearman correlations was not possible

Mice treated with	Th2 versus IL-10	
	r	*P*
P0	-	-
P1	0.49	0.18
P2	0.65	0.05
P0-(P1-P2)$_2$	0.82	0.006
Msp-1$_{19}$	0.81	0.007
adjuvant control mice	0.15	0.54
untreated control mice	0.21	0.41

Table S5.

Production of IL-10 is associated with increased Th2 lymphocyte numbers. The Spearman correlation between the numbers of serum IL-10 and Th2 (CD4+CD30+) lymphocytes in the peripheral blood of mice immunized with the P0, P1, P2, P0-(P1-P2)$_2$, and Msp-1$_{19}$ proteins and in the adjuvant as well as the untreated control mice. Correlations were calculated in each study group with eighteen mice. Values of $P \leq 0.05$ were considered significant.

- the concentrations of circulating IL-10 levels in mice immunized with the P0 protein were below the detection threshold; therefore, calculation of Spearman correlations was not possible

Mice treated with	total IgG versus Th2	
	r	*P*
P0	0.65	0.056
P1	0.37	0.33
P2	0.68	0.04
P0-(P1-P2)$_2$	0.91	0.0006
Msp-1$_{19}$	0.89	0.001
adjuvant control mice	0.21	0.26
untreated control mice	0.2	0.33

Table S6.

Humoral immune response support. The Spearman correlation between the IgG levels and the abundance of Th2 lymphocytes in the peripheral blood of mice immunized with the P0, P1, P2, P0-(P1-P2)$_2$, and Msp-1$_{19}$ proteins. For IgG determination, sera in each mouse group were pooled (n=9). Values of $P \leq 0.05$ were considered significant.

Mice immunized with	IL-10 versus:			
	total IgG		Th2	
	r	*P*	r	*P*
P0	-	-	-	-
P1	0.96	0.0004	0.49	0.18
P2	0.95	0.0006	0.65	0.057
P0-(P1-P2)$_2$	0.69	0.037	0.82	0.006
Msp-1$_{19}$	0.66	0.049	0.82	0.007
adjuvant control mice	0.19	0.29	0.22	0.39
untreated control mice	0.09	0.39	0.28	0.43

Table S7.

IL-10 promotes humoral response. The Spearman correlation between IgG levels versus IL-10 serum concentrations and Th2 lymphocyte numbers in the peripheral blood of mice immunized with the P0, P1, P2, P0-(P1-P2)$_2$, and MSP-1$_{19}$ proteins and in the adjuvant control mice as well as the untreated control mice. For measurements of both IgG and IL-10, sera in each mice group were pooled (n=9). P0 did not induce detectable production of serum IL-10. Values of $P \leq 0.05$ were considered significant.
